# The role of autologous bone marrow transplantation in primary effusion lymphoma: a case report and literature review

**DOI:** 10.1016/j.htct.2024.04.119

**Published:** 2024-07-23

**Authors:** Vitor Abreu de Goes, Anita Cassoli Cortez, Diogo Lago Morbeck, Felipe D'Almeida Costa, Talita Bueno da Silveira

**Affiliations:** a Albert Einstein Israeli Faculty of Health Sciences, São Paulo, SP, Brazil; b A.C.Camargo Cancer Center, São Paulo, SP, Brazil

**Keywords:** Extracavitary primary effusion lymphoma, HHV8, Primary effusion lymphoma, Autologous bone marrow transplantation

## Abstract

Primary effusion lymphoma (PEL) is an aggressive and rare type of diffuse large B-cell lymphoma (DLBL) that commonly presents itself as pleural, pericardial or peritoneal effusion without lymph node or extranodal involvement in immunosuppressed patients, such as HIV-positive or transplanted receptors. On rare occasions, it may be found in solid sites without effusion, in an immunophenotypically and morphologically similar neoplasm well-known as extracavitary PEL (EC-PEL). Both PEL and EC-PEL are associated with extremely poor prognosis. Due to the rarity of these entities, ther e are no gold standard treatments . Here we discuss the role of autologous bone marrow transplant (auto-BMT) in the treatment of these patients as well as report the case of a young HIV-positive male diagnosed with both PEL and EC-PEL, who underwent a salvage therapy with auto-BMT and achieved complete and sustained remission eight years after the diagnosis.

## Introduction

Primary effusion lymphoma (PEL) is an aggressive and uncommon type of diffuse large B-cell lymphoma, usually associated with an extremely poor prognosis. Although it may be present in other immunologically compromised populations such as the elderly and transplant recipients, it mainly affects patients with a history of HIV infection.^1^, ^2^, ^3^ The estimated lifetime prevalence of non-Hodgkin lymphoma (NHL) lies from 5 % to 20 %, of which PEL makes up about 4 %.^1^^,^^4^^,^^5^

The most common clinical presentation is cavitary effusions with no lymphadenopathy. PEL may also exhibit rarer presentations, such as multicavitary involvement or the presence of solid tumor masses. In the latter, the neoplasm shows great similarity to PEL both morphologically and immunophenotypically, which guaranteed its recognition as a variant of its own named extracavitary PEL (EC-PEL).^6^^,^^7^ In contrast with PEL, EC-PEL tends to affect the intestinal tract, skin, lungs, central nervous system and lymph nodes and may be found at the time of diagnosis of PEL.^4^

Morphologically both pathologies appear to carry immunoblastic, plasmablastic and anaplastic lymphomas, with cells mainly presenting large and irregular nuclei and moderate to abundant basophilic cytoplasms but also having poorly defined perinuclear hofs that resemble Reed-Sternberg cells in classical Hodgkin lymphoma.^2^^,^^3^^,^^6^ Although having a B-cell origin, both PEL and EC-PEL often express CD45, CD30, CD38 and MUM1 and lack pan-B cell antigens such as CD20 and CD79a. EC-PEL tends to express more CD20, CD79a and CD3 and less CD45 when compared to the classic variant, but in general their phenotypes are quite similar.^1^^,^^3^^,^^5^

As EC-PEL was only recently described in the literature, it is difficult to compare its prognosis with classic PEL, since antiretroviral therapy (ART) was less effective in older studies than it is nowadays. The prognosis of the newer series has improved but it still remains unsatisfactory, with a median overall survival of 10.2 months and a five-year overall survival (OS) rate ranging from 39 % in EC-PEL to 43 % in classic PEL.^3^^,^^5^^,^^7^

Given its rarity, there is currently poor understanding of the disease pathogenesis and most of the available knowledge is based on case reports or series. When diagnosed, these cancers are treated with regimens intended for other non-Hodgkinʼs lymphoma, such as cyclophosphamide, doxorubicin hydrochloride, vincristine sulfate, and prednisone (CHOP)-like regimens alone or associated with high-dose methotrexate (MXT), both with unsatisfactory results. As PEL appears to be a monoclonal lymphoproliferative disease, autologous stem cell transplant may be an option for younger patients with relapsed or refractory PEL as presented below.^8^^,^^9^

## Methods

A literature review, performed in an academic medical setting, searched for cases of PEL and EC-PEL submitted to autologous bone marrow transplantation (auto-BMT) reported between 2002 and 2022. Epidemiological data such as patients' age, first line treatment, salvage treatment, Epstein–Barr (EBV) status, disease status before auto-BMT, conditioning regimen and outcome were extracted. The literature search was conducted on PubMed/MEDLINE, Web of Science, Scopus, EMBASE and Cochrane Library, using the search terms ‘primary effusion lymphoma’ AND ‘autologous bone marrow transplantation’.

Three articles that met criteria for this review were found: six patients are described in Table 1. Two articles reported cases with relapsed PEL treated with second-line auto-BMT whereas the other one reported four cases of patients with PEL treated with first-line auto BMT.

**Table 1:** Patients with primary effusion lymphoma treated with autologous bone marrow transplantation from 2002 to 2022.

Author	Year	Age	EBV	First-line treatment	Salvage treatment	Disease status prior to auto-BMT	Conditioning Regimen	Outcome
**First line treatment with Auto-BMT**								
Mirza, AS et al.	2019	62	positive	Hyper- CVAD	–	CR	BEAM	CR at 23 months after BMT
Mirza, AS et al.	2019	33	NA	CHOP	–	PD	BEAM	Relapse 3 mo after BMT
Mirza, AS et al.	2019	39	positive	DA-EPOCH	–	CR	BEAM	Relapsed 6 months after BMT; Died 9 months after BMT
Mirza, AS et al.	2019	49	positive	Hyper-CVAD	–	CR	BEAM-R	CR at 20 months after TMO
**Salvage treatment with Auto-BMT**								
Waddington TW et al.	2004	48	positive	3 cycles of EPOCH	1 cycle of ICE	NA	BuCy	Died 2 weeks after BMT
Won, JH et al.	2006	62	negative	6 cycles CHOP	3 cycles of ICE	CR	BEAM	CR at 12 months after BMT

Auto-BMT: Autologous bone marrow transplantation; BEAM: Busulfan and etoposide; BEAM-R: BEAM plus rituximab; BMT: Bone marrow transplantation; BuCy: Busulfan, cyclophosphamide, cytarabine, and melphalan; CHOP: Cyclophosphamide, adriamycin, vincristine and prednisone; CR: Complete remission; EPOCH: Etoposide prednisone vincristine cyclophosphamide and adriamycin; F-GIV: Ifosfamide plus inorelbine, gemcitabine and pegfilgrastim; ICE: ifosfamide, cyclophosphamide and etoposide; NA Not available; PD Progressive disease.

## Case description

A 48-year-old Caucasian male was referred to A.C. Camargo Cancer Center in May 2013 due to a right leg skin biopsy compatible with Kaposi's sarcoma (KS). Immunohistochemistry showed desmin markers, smooth muscle actin, S-100 protein-negative neoplastic cells, and CD34 and HHV8 were positive in neoplastic cells with a 10 % Ki 67. He had been under investigation for the previous three months concerning right lower limb edema and inguinal adenomegaly, with an ultrasound before admission displaying a 3.3 cm lymph node for which a fine-needle aspiration biopsy was inconclusive. There was no history of fever, sweating, pruritus, or weight loss during this period. At the time of the KS diagnosis, the HIV serology was positive, with a CD4 lymphocyte count of 75 cells/µL at diagnosis, and so ART (lamivudine, efavirenz, and tenofovir) was initiated.

Abdominal and thoracic computed tomography (CT) were performed to stage Kaposi's sarcoma, as was an esophagogastroduodenoscopy (EGD) and a colonoscopy. These exams revealed metastasis at bronchi, pleura, stomach, rectum, lymph nodes and at the tongue base, which were further confirmed by biopsies.

The chemotherapy regimen proposed was six cycles of paclitaxel 100 mg/m². One month after therapy ended, the patient experienced pleural effusions. This was analyzed by conventional cytopathology together with a paraffin embedded cell block that show a predominance of areas of granulation tissue associated with chronic inflammatory infiltrate, with scattered atypical cells with large eccentric vesicular nuclei, prominent nucleoli and a moderate amount of basophilic cytoplasm. The immunohistochemistry performed revealed CD45/LCA, CD138, HHV8 positive neoplastic cells with lambda chain restriction and Ki 67 positivity in 100 % of atypical cells. The search for EBV using the chromogenic *in situ* hybridization (CISH) method was positive for the cells of interest. The findings corroborated the hypothesis of pleural PEL.

**Figure 1:**
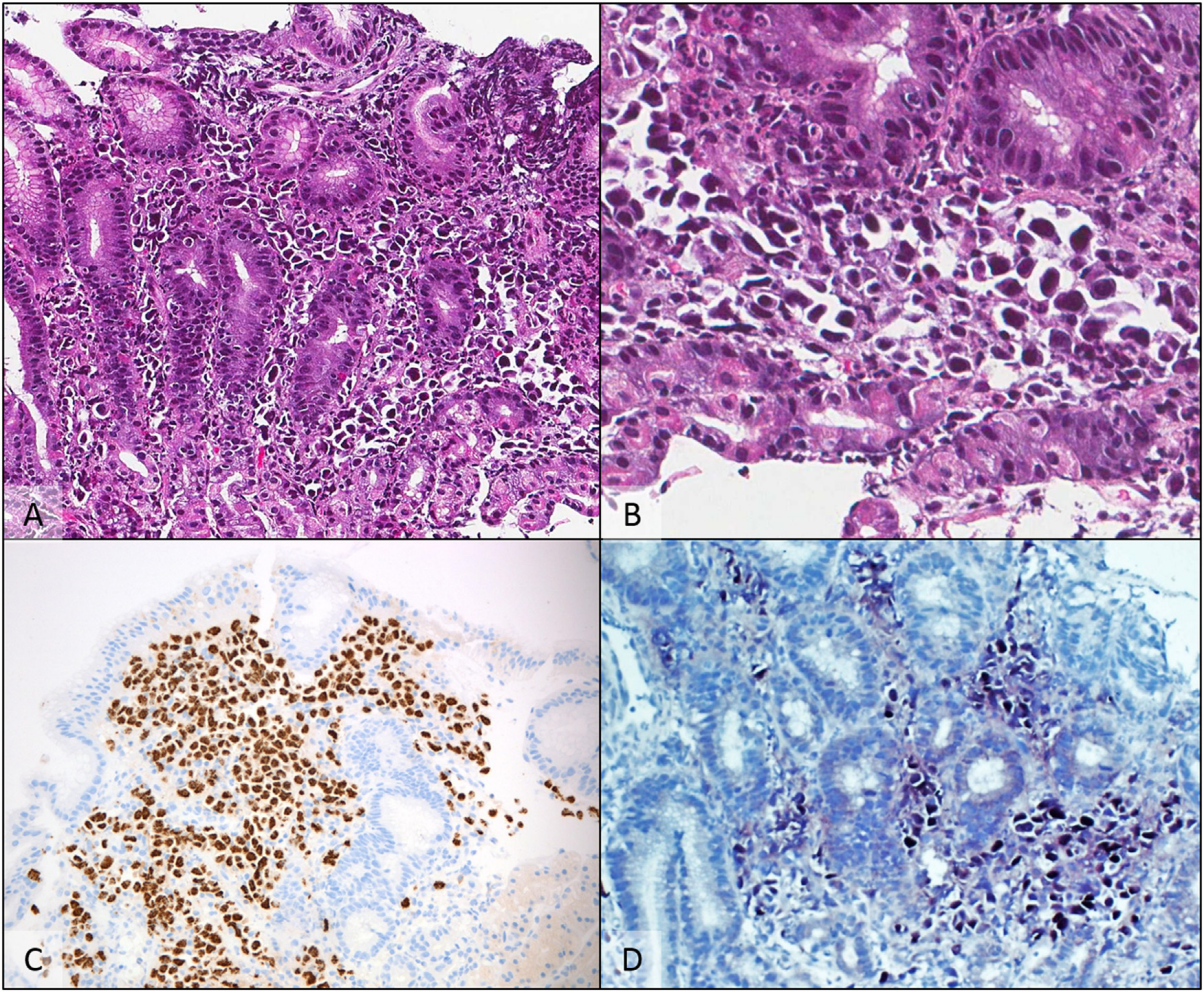
A gastric biopsy showed numerous atypical cells replacing the lamina propria A: (H & E - 200×). At a higher power, cells were discohesive, with eccentric nuclei and deeply basophilic cytoplasm B: (H & E - 400×). Immunohistochemical studies showed diffuse positivity for HHV-8 C (anti-HHV-8 - 200×), CD138 and MUM-1 (not shown), with EBV-encoded RNA detected by chromogenic *in situ* hybridization D (EBER CISH - 200×).

**Figure 2:**
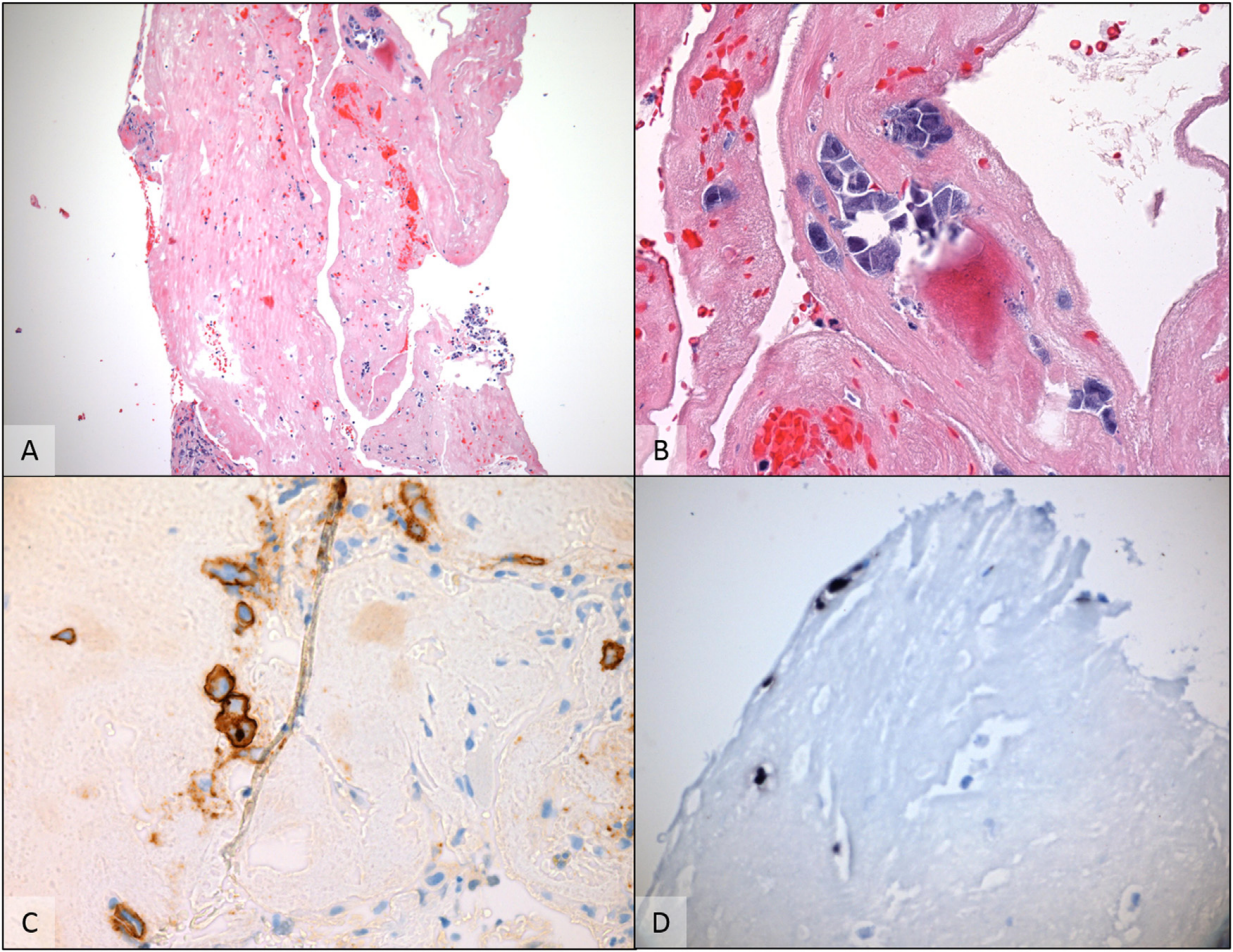
A cell block preparation from the pleural effusion revealed scattered atypical cells floating around and within fibrinous material A (H & E - 100×). The cells had plasmablastic appearance, with large eccentric vesicular nuclei, prominent nucleoli and basophilic cytoplasm B (H & E - 400×). By immunohistochemically, cells were positive for CD138 C (anti-CD138 - 400×) and HHV-8 (not shown). Chromogenic *in situ* hybridization for EBV- encoded RNA was also positive in this specimen D (EBER CISH - 400×) supporting the diagnosis of Primary effusion lymphoma.

The patient then received a six-cycle treatment of CDE (cyclophosphamide 200 mg/m^2^ for 4 days; doxorubicin 12.5 mg/m^2^ for 4 days; etoposide 60 mg/m^2^ for 4 days) combined with intrathecal chemotherapy with complete remission (CR) being documented by positron emission tomography (PET). The patient stayed in remission for seven months until a follow-up EGD revealed multiple lesions throughout the gastric wall and one lesion in the anterior segment of the duodenum; biopsies revealed fragments of gastric mucosa with relatively preserved foveolar epithelium. The lamina propria was infiltrated by large atypical cells with plasmablastic morphology and large eccentric vesicular nuclei, prominent nucleoli and moderate amount of basophilic cytoplasm. By immunohistochemically, these cells were positive for CD138, MUM-1, HHV-8 and EBER and negative for ALK-1, Bcl2, Bcl6, CAM5.2, CD3, CD5, CD10, CD20, CD23, CD30, CD34 and Cyclin D, confirming its plasmablastic nature and the diagnosis of gastric involvement by PEL (Figures 1 and 2).

Rescue chemotherapy was chosen with three cycles of the gemcitabine, dexamethasone and cisplatin (GDP) scheme with CR occurring after three cycles. The patient then underwent an auto-BMT using the Busulfan/Cyclophosphamide/Etoposide (BuCyE) regimen 14 months after the diagnosis of PEL. He maintained CR on CTs performed two and seven months post-transplant and EGD performed 13 months post-transplant. He has been followed up each six months with neither clinical nor laboratory findings of lymphoproliferative disease for eight years after diagnosis.

**Histopathology**

## Discussion

Given its rarity and refractoriness to chemotherapy, a standard treatment for PEL has not yet been identified. According to the current National Comprehensive Cancer Network (NCCN) guidelines, the use of chemotherapy regimens such as R-DA-EPOCH (rituximab, etoposide phosphate, prednisone, vincristine sulfate, cyclophosphamide, and doxorubicin hydrochloride), rituximab-CHOP, CODOX-M/IVAC (cyclophosphamide, vincristine, doxorubicin, high-dose MXT, ifosfamide, etoposide and high-dose cytarabine) and R-HyperCVAD (rituximab, cyclophosphamide, vincristine sulfate, doxorubicin hydrochloride, and dexamethasone) combined with ART is recommended for AIDS-related B-cell lymphoma. High-dose MTX seems to be an inferior choice as it increases treatment toxicity while lacking differences in OS and progression-free survival (PFS).^1^^,^^7^^,^^10^ As the association of etoposide with CHOP-like regimens proved slightly beneficial in the treatment for NHL in some case series, we opted for the CDE regimen as the first-line treatment.^11^, ^12^, ^13^

Although PEL and EC-PEL apparently do not present significant differences in terms of population profiles and prognosis, little is known about patients with both conditions. It is known that more recent reports of PEL and EC-PEL have a better prognosis when compared to older series, with one crucial factor being the improvement of combination ART, which drastically altered the natural history of AIDS-related lymphoma (ARL).^7^^,^^14^ Another factor is the lower HIV-associated morbidity in patients with ARL that allows greater tolerance to therapies, including autologous stem cell transplantation. In a German multicenter cohort study, effective viral replication control was shown to be independently associated with improved survival in patients with ARL,^15^ while adding HIV related variables was shown to increase the predictive value of the International Prognostic Index for risk of death.^16^ This is in agreement with a report by Boulanger et al. that shows that the only independent prognosis indicator, other than an impaired performance status, was the absence of preexisting combination ART use.^10^ The patient in our case had a CD4 count above 50 cells/µL at the time of diagnosis and undetectable viral load for the previous two years, which might be a contributing factor to the better outcome.

The role of the co-infection by EBV in PEL is not entirely understood yet, even though 80 % of classic PEL cases are EBV positive.^17^ A series report with 51 PEL and EC-PEL HIV-positive patients was unable to find a correlation between EBV-positive PELs and OS.^7^

Our literature survey revealed that only a small number of studies regarding high-dose chemotherapy with auto-BMT in PEL or EC-PEL patients have been published, with a total of six patients apart from this report.^8^^,^^9^^,^^17^ Four patients received auto-BMT as first-line therapy in a study by Mirza AS et al. All underwent transplantation after reaching first CR with high-dose chemotherapy. The two-year PFS and OS for patients who had received auto-BMT was 50 % and no deaths were attributable to auto-BMT at two years after autografting.^18^

The last two patients reported received auto-BMT as salvage therapy. The current case concerns a sustained CR after an auto-BMT, which is similar to the case report published by Won et al. regarding a 62-year-old HIV-negative patient.^8^ He presented pericardial effusion compatible with PEL and achieved CR after six cycles of CHOP and sustained remission for 12 months after rescue auto-BMT. In contrast, Waddington et al. reported a case of a 48-year-old patient who underwent first-line treatment with EPOCH (etoposide, prednisone, vincristine, cyclophosphamide and doxorubicin hydrochloride) and salvage auto-BMT who died a few weeks after the stem cell infusion due to disease progression. It should be noted that this patient did not achieve CR after the first-line regimen and his status prior to auto-BMT is not clear.^9^

The role of auto-BMT in PEL is still very controversial. Its use was described by Hubel et al. as an option early on during treatment in order to avoid resistance to chemotherapy.^19^ However, no clear outcome distinctions were found between the articles in our literature review given the small number of cases due to the rarity of this pathology.

## Conclusion

As both PEL and EC-PEL are extremely rare pathologies, and so clinical trials are not feasible, these conditions are still treated with regimens intended for other non-Hodgkin's lymphoma, with unsatisfactory results. A better understanding of the pathophysiology and clinical behavior of PEL and EC-PEL through case and series reports is essential for seeking new options in the therapeutic management of these patients. Auto-BMT may be a possible treatment for some patients, even though the results in the literature are heterogeneous and our impressions are far from definitive. Here we report on a case of a successfully treated patient after bone marrow transplantation as salvage therapy who stayed in CR during an eight-year follow-up.

## Conflicts of interest

The authors declare no conflicts of interest
